# Soybean Endo-β-Mannanase GmMAN1 Is Not Associated with Leaf Abscission, but Might Be Involved in the Response to Wounding

**DOI:** 10.1371/journal.pone.0049197

**Published:** 2012-11-16

**Authors:** Min Yan, Yifan Zhang, Wenjuan Guo, Xiaofeng Wang

**Affiliations:** College of Life Sciences, South China Agricultural University, Guangzhou, People’s Republic of China; University of Massachusetts Amherst, United States of America

## Abstract

The objective of this work is to investigate the relationship between endo-β-mannanase and leaf abscission, and response to wounding in soybean (*Glycine max*). An endo-β-mannanase gene *GmMAN1* was cloned from the abscission zone in petiole explants, and was heterologously expressed in *E. coli*. Polyclonal antibodies were raised against the fusion protein. The increases in activity, isoform numbers, and amounts of transcripts and proteins of *GmMAN1* were found not only in the abscission zone but also in the non-abscission zone during petiole abscission in the explants, but not in these two tissues during leaf abscission artificially induced by ethephon treatment in the intact plants. The changes in endo-β-mannanase expression patterns in these two tissues were probably induced by the inherent mechanical wounding during the preparation of explants. When soybean plants were wounded by removing half of the leaf blade of the first pair of true leaves, the transcripts and proteins of *GmMAN1* were induced in the leaves and stem, leading to the increases in enzyme activity and isoform numbers in them. It is concluded that the soybean endo-β-mannanase GmMAN1 is not associated with leaf abscission, but might be involved in the response to wounding.

## Introduction

Endo-β-mannanase (EC 3.2.1.78) hydrolyzes the β-1, 4-linkages in the backbone of mannans, one of plant cell wall hemicelluloses. This enzyme plays an important role in plant growth and developmental events in which cell wall degradation is involved. For example, loosening of the micropylar endosperm cell walls, a prerequisite for the completion of seed germination in tomato, *Datura ferox* and *Arabidopsis thaliana* is accompanied by an increase in endo-β-mannanase activity [1]–[3]. The post-germination mobilization of mannans, largely stored in the endosperm cell walls of some legume seeds such as *Trigonella foenum-graecum* requires the activity of endo-β-mannanase [4]. Tomato fruit softening and pollen tube elongation, in which cell wall remodeling happens, also need the action of endo-β-mannanase [5], [6]. Organ abscission and response of plants to wounding are other growth and developmental events that involve cell wall breakdown [7]–[10]. Therefore, it is of interest to know if endo-β-mannanase also plays a role in organ abscission and response of plants to wounding.

Abscission is the process whereby organs are shed from the plant. A characteristic of abscission is the synthesis of hydrolases leading to the breakdown of cell walls of separation layer and finally the shedding of organs [8]–[10]. A number of cell wall-degrading enzymes are reported to be associated with abscission. For example, the activity of cellulase (β-1,4-glucanase) increases in the abscission zone (AZ) during the shedding of leaves, flowers and pods of soybean [11], [12], leaves of *Phaseolus vulgaris* [13], leaflets of *Sambucus nigra* [14], flowers of tomato [15] and flowers and leaves of *Capsicum annuum* [16]. Polygalacturonase activity increases in the AZ during the shedding of leaves [17], [18], flowers [18], [19] and fruits [12], [20]. Other pectin-degrading enzymes, including pectin methylesterase and pectate lyase also play a probable role in the leaf abscission of soybean [21] and citrus [22]. The roles of some hemicellulose-degrading enzymes in organ abscission also have been reported. For example, the expression of xyloglucan endotransglycosylase/hydrolase genes is up-regulated in the leaf AZ of soybean [21] and citrus explants [22]. β-Galactosidase is overrepresented in the citrus leaf AZ during abscission [22]. In addition to cell wall-degrading enzymes, expansins, a group of plant cell wall proteins which do not exhibit hydrolytic or other enzymatic activity but can loosen cell walls by disrupting hydrogen bonds between polysaccharides [23], also play a role in the abscission of *Sambucus nigra* leaflets [24], soybean leaves [21] and rose petals [25]. A cocktail of hydrolytic enzymes or proteins in addition to the above may contribute to the degradation of cell walls that accompanies the shedding of plant organs [8]–[10]. Therefore, endo-β-mannanase, a hemicellulose-degrading enzyme, may also play a role in organ abscission. But to our knowledge this has not been reported.

Wounding of plants induces a series of responses, including the systemic synthesis of cell wall-remodeling enzymes, such as pectinase [7], β-1,3-glucanase [7], [26]–[28], and polygalacturonase [29], [30]. These enzymes are able to protect the plants from infection by attacking the cell wall of invading fungi and/or to release endogenous oligosaccharides (elicitors) from the plant cell walls to further stimulate the defense response [7]. For example, oligogalacturonic acid fragments, the products of polygalacturonase action, can trigger the accumulation of H_2_O_2_ which is a response to wounding in a number of plant species [29]. Oligosaccharides derived from spruce galactoglucomannan have been shown be able to induce non-specific resistance to local tobacco necrosis virus infection in cucumber [31]. Therefore, endo-β-mannanase, capable of producing mannooligosaccharides, may be involved in the response of plants to wounding, although there is no report of this.

In the present work, an endo-β-mannanase gene was cloned from the AZ of soybean petiole explants, polyclonal antibodies were raised against its encoded protein. The expression patterns of activity, isoforms, proteins and transcripts of this enzyme were analyzed during petiole abscission in the explants and leaf abscission artificially induced by ethephon treatment in the intact plants. Then, the changes in the expression patterns of this enzyme were examined in the leaves, stem and roots of soybean plants to which leaves were wounded.

## Materials and Methods

### Ethics Statement

All rabbits were raised under standardized pathogen-free conditions in the College of Veterinary Medicine at South China Agricultural University. The study protocol for the experimental use of the animals was approved by the Ethics Committee of South China Agricultural University.

### Plant Materials and Treatments

Seeds of soybean (*Glycine max*, cv. Wujiangqingdou) were surface sterilized in 10% (v/v) H_2_O_2_ for 1 min, washed with distilled water and germinated in clean sand irrigated with 1/4 strength Hoagland nutrient solution in darkness at 25°C. After the first pair of true leaves had fully expanded and the first trifoliolate leaf had just emerged (c.a. 7 d from sowing), seedlings were transplanted into 40 L tanks with a nutrient solution composed of 1.5 mM KNO_3_, 1.2 mM Ca(NO_3_)_2_, 0.4 mM NH_4_NO_3_, 0.5 mM MgSO_4_, 0.3 mM K_2_SO_4_, 0.3 mM (NH_4_)_2_SO_4_, 25 µM MgCl_2_, 5 µM Fe-EDTA(Na), 1.5 µM MnSO_4_, 1.5 µM ZnSO_4_, 0.5 µM CuSO_4_, 0.15 µM (NH_4_)_6_Mo_7_O_24_ and 0.5 µM NaB_4_O_7_ in a greenhouse at the Department of Plant Nutrition, South China Agricultural University at an average temperature of 28°C/19°C (day/night). The solution was well aerated and the pH was maintained between 5.8 and 6.0 with daily addition of 1.0 mM KOH or HCI.

**Figure 1:**
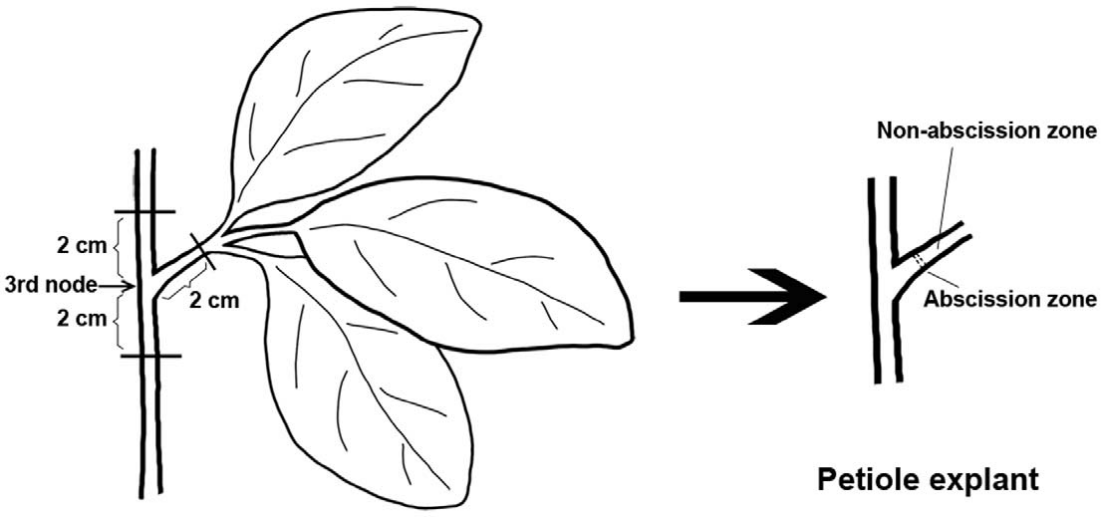
Diagram illustrates the location of soybean petiole explants.

Petiole explants for the experiment of abscission were excised from soybean plants 25 d after the transplanting of seedlings from sand to tanks with a nutrient solution. To maximize uniformity, all explants were excised from the third node by cutting the stem at 2 cm above and below the node and cutting the petiole at 2 cm from its base as illustrated in Figure 1. The explants containing only the proximal AZ were surface-sterilized in 0.5% (w/v) sodium hypochlorite for 5 min, rinsed with several volumes of distilled water and finally placed upright in wet sand and maintained in a sealed container in the dark at 25±1°C. Petiole abscission was determined if they were detached by a light touch with the forceps and was recorded every 12 h. The cumulative abscission percentage was calculated as (number of petioles detached/total number of petioles) × 100. Leaf AZ (1 mm on either side of the fracture plane) and non-abscission zone (NZ) (5 mm away from AZ) were harvested, frozen immediately in liquid nitrogen and stored at −80°C until analysis.

Leaf abscission was also artificially induced in intact soybean plants 25 d after transplanting by spraying 0.1% (v/v) ethephon+0.05% (v/v) Tween-20. The intact plants of same age sprayed with water were used as a control. A second spray was applied 1 d after the first. Leaf abscission was recorded every 24 h and cumulative abscission percentage was calculated as above. The proximal AZ and NZ were harvested, frozen immediately in liquid nitrogen and stored at −80°C until analysis.

For clarity, the tissues examined in petiole explants and intact plants are listed in Table 1.

**Table 1:** Tissues examined (√) in petiole explants and intact plants during abscission process.

Tissues examined	Petiole explants	Intact plants	
		Treated with ethephon	Treated with water as a control
Abscission zone	√	√	√
Non-abscission zone	√	√	√

The wounding experiment was carried out by using soybean plants 10 d after the transplanting of seedlings from sand to tanks with a nutrient solution. Plants were wounded by removing half of the leaf blade of the first pair of true leaves. After wounding, the roots, stem and remaining leaf blades were harvested every 12 h, immediately frozen in liquid nitrogen and stored at −80°C until analysis. The tissues examined in wounded and non-wounded (control) plants were listed in Table 2.

**Table 2:** Tissues examined (√) in wounded and non-wounded soybean plants.

Tissues examined	Wounded plants	Non-wounded plants as a control
Root	√	√
Stem	√	√
Leaf	√	√

### Extraction, Activity Assay and Isoform Detection of Endo-β-mannanase

The AZ and NZ tissues in petiole explants and intact plants, or the roots, stem and remaining leaf blades in wounded and non-wounded (control) plants were ground in an ice-cold mortar in 0.1 M Hepes-NaOH buffer (pH 8.0). The volume of the buffer was added at the ratio of fresh weight of tissues (mg):volume of buffer (mL) = 1∶3. The extract was centrifuged at 4°C for 10 min at 13,000 g, and the supernatant was used for activity assay and isoform detection of endo-β-mannanase as previously described [32].

### Cloning of *GmMAN1* cDNA

About 0.1 g of the AZ tissue was pulverized in liquid nitrogen and extracted in 1 mL of TRIzol reagent (Invitrogen, Carlsbad, USA) for total RNA. First-strand cDNA was synthesized from 1–3 µg of total RNA using the primer (5′-ACATGAGCTAGGTAGGCGACATTA) and an M-MLV RTase cDNA Synthesis Kit (TaKaRa, Dalian, China) according to the manufacturer’s instructions. Using the reverse transcription product as template, PCR was performed with the specific forward (5′-CTCTTCTCTGCCGTCCAAGCTA) and reverse (5′-ACATGAGCTAGGTAGGCGACATTA) primers. These were designed from a sequence electronically cloned using the tomato endo-β-mannanase gene *LeMAN1* (GenBank ID: AF017144) to do BLAST analyses against a soybean EST library. The PCR procedure was as follows: 94°C for 4 min followed by 30 cycles of 94°C for 1 min, 57°C for 1 min, and 72°C for 90 s. The final cycle was followed by extension at 72°C for 10 min. The amplified cDNA was ligated to the pGEM-T Easy vector (Promega, Madison, USA), and sequenced.

### Expression of Recombinant Protein in *E. coli*

The coding region (without the signal peptide) of the *GmMAN1* cDNA (bp 85–1,233) was amplified by PCR using an *Eco*RI site-linked forward primer (5′-AAAAGAATTCATGGGTTGGAAAGGAGGTCTCAA) and an *Xba*I site-linked reverse primer (5′-AAAATCTAGACATTAATGCTTGTTCACTAAGCA). The product was digested with *Eco*RI and *Xba*I and ligated into the *Eco*RI and *Xba*I sites of the maltose-binding protein expression vector pMAL-c2X (New England Biolabs, Beverly, MA). The empty vector and the vector containing insertion were transformed into competent cells of *E.coli*, strain DH5α. Selection and incubation of the resulting transformant cells and induction, extraction, examination and purification of the expressed fusion proteins were carried out as previously described [33], [34].

### Preparation of Polyclonal Antibodies

An adult male rabbit was subcutaneously injected with a mixture of a solution containing 1–2 mg purified fusion protein and a same volume of complete Freund’s adjuvant (Sigma). After 10 d, two more injections were carried out with the same dosage of Freund’s incomplete adjuvant (Sigma) at intervals of 7 d. Two weeks after the last injection, the antiserum was collected from the rabbit’s carotid artery.

### SDS-PAGE and Immunoblotting

The protein content of each enzyme extract was determined using the method described by Bradford [35]. Proteins (100 µg) were separated by SDS-PAGE using 10% (w/v) polyacrylamide separation gels. After electrophoresis, proteins were transferred to PVDF membranes (Millipore, Billerica, MA), which were blocked with 3% (w/v) BSA in TTBS (Tween-Tris buffered saline) for 2–4 h at room temperature. The membranes were washed five times with TTBS each for 5 min before transfer to the first antibody solution in 3% (w/v) BSA in TTBS for 1–2 h. Antibodies developed as above were used for immunoblotting at a 1∶500 dilution. Bound antibody was detected using alkaline phosphatase-conjugated goat anti-rabbit immunoglobulin (Sigma-Aldrich, The Netherlands). The bands were detected after the reaction with the Detection Reagents NBT (Sigma) and BCIP (Sigma).

### RNA Gel Blot Analysis

Total RNA (20 µg) extracted from the AZ and NZ tissues in petiole explants and intact plants, or from the roots, stem and remaining leaf blades in wounded and non-wounded (control) plants was subjected to electrophoresis as described previously [32]. The cDNA probes designed from the sequences at 3′-end of the open reading frame of *GmMAN1*, including an 18-bp 3′-UTR, were prepared using the specific forward (5′-TGTTGACAAATGGTTACAAACA) and reverse (5′-GACATTAATGCTTGTTCACTAAGCA) primers according to the manufacturer’s instructions for a Random Primer DNA Labeling Kit (TaKaRa, Dalian, China) using [α-^32^P] dCTP. The membranes were prehybridized, hybridized and washed as described previously [32]. The hybridization signal was detected and analyzed using the Molecular Imager FX system and Quantity One software (Bio-Rad, Hercules, CA, USA).

## Results

### Changes in Endo-β-mannanase Activity and Isoform Profiles during Petiole Abscission in Explants

**Figure 2:**
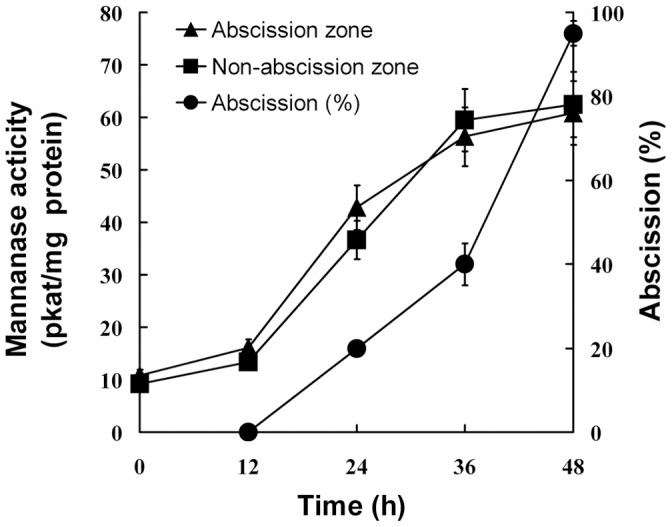
Changes in the cumulative abscission percentage and endo-β-mannanase activity in abscission and non-abscission zones of soybean petiole explants. Means of three measurements ±SD.

To determine whether endo-β-mannanase is involved in leaf abscission, petiole explants were excised from 32-d-old soybean plants. Petioles began to abscise from 36 h after excising (Figure 2). The cumulative abscission percentage increased to 30% at 60 h and reached a maximum of 80% by 72 h.

Endo-β-mannanase activity was assayed in the AZ and NZ tissues during petiole abscission in explants (Figure 2). It was detectable at a level of c.a. 10 pkat/mg protein in both the AZ and NZ in freshly-excised explants (0 h). Up to 36 h, the activity in both the AZ and NZ increased gradually, although no petiole abscised during this time. After the petioles started to abscise (later than 36 h), the enzyme activity kept increasing until 72 h. However, the enzyme activity in the AZ was not significantly higher than that in the NZ during the whole process.

**Figure 3:**
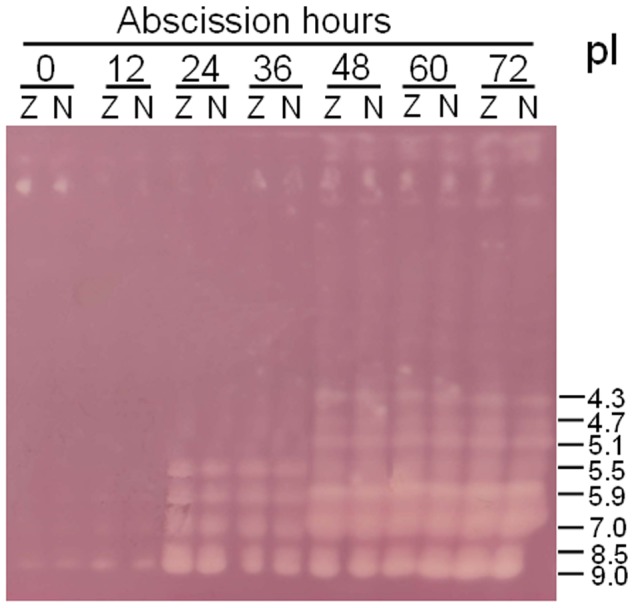
Changes in the ultrathin-layer isoelectric focusing isoform profiles of endo-β-mannanase in abscission zone (Z) and non-abscission zone (N) of soybean petiole explants. Isoelectric points (pI) are indicated on the right. Equal amounts (30 µg) of protein were loaded on each lane.

To determine if the isoform profiles of endo-β-mannanase were different between the AZ and NZ, enzymes extracted from the two tissues were analyzed by ultrathin-layer isoelectric focusing (Figure 3). The results showed that one isoform (pI 9.0) existed constitutively in freshly-excised petiole explants. After 24 h, four new isoforms (pI 8.5, 7.0, 5.9 and 5.5) were detected. Three more isoforms (pI 5.1, 4.7 and 4.3) appeared from 48 h after excising. However, like enzyme activity, there was no difference of endo-β-mannanase isoform numbers between the AZ and NZ tissues at each time point examined.

### Isolation of Soybean *GmMAN1* cDNA from Abscission Zones in Petiole Explants

To analyze the expression patterns of endo-β-mannanase at the transcriptional level during petiole abscission in explants, a gene encoding this enzyme was cloned from the AZ. For this, the tomato endo-β-mannanase gene *LeMAN1* [36] was used for BLAST analyses against a soybean EST library. One contig of 1441-bp was electronically cloned from the homologous soybean EST sequences, and specific primers were designed from this. A soybean endo-β-mannanase cDNA was obtained by reverse transcriptase (RT)-PCR and the sequence (Figure S1) was registered in the GenBank as accession DQ812101. Recently, Lin *et al.* cloned an endo-β-mannanase gene from soybean seed and termed it as *GmMAN1* [37]. We found that our cDNA sequence was similar to that documented by Lin et al. [37], only with several different nucleotides at some positions. This is probably due to different cultivars were used by two groups or originated from the cloning procedures (e.g., reverse transcription and/or PCR amplification). Thus the cDNA we cloned from the AZ was *GmMAN1*. The open reading frame of *GmMAN1* is 1233-bp in length, encoding 410 amino acids with the first 28 amino acids as a putative signal peptide. After removal of the signal peptide, the protein has a molecular mass of 43 kDa and a pI of 7.01. The deduced amino acid sequence of *GmMAN1* showed a similarity to other plant endo-β-mannanase genes, such as a 61% identity with *manA* of *Coffea arabica* [38], 56% with *LsMan1* of *Lactuca sativa* [39] and 54% with *LeMAN1* of *Lycopersicon esculentum* [36].

### Expression Time Course of *GmMAN1* during Petiole Abscission in Explants

**Figure 4:**
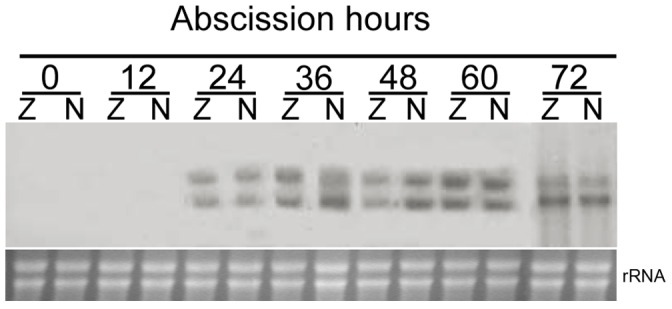
Changes in the abundance of *GmMAN1* transcripts in abscission zone (Z) and non-abscission zone (N) in soybean petiole explants. Equal amounts (20 µg) of RNA were loaded on each lane.

Total RNA was extracted from the AZ and NZ tissues of soybean petiole explants during abscission, and used for RNA gel blot analysis (Figure 4). Up to 12 h after excising, no signal was detectable in the AZ and NZ tissues. From 24 h, two mRNA bands of close molecular mass were detected in both the AZ and NZ tissues using the cDNA probe for *GmMAN1*. This is probably another member of soybean endo-β-mannanase gene family with high sequence identity to *GmMan1* was also recognized by the probe used. The abundance of the two bands in both the AZ and NZ tissues remained almost unchanged after 24 h. In addition, no difference in the abundance of the two bands was observed between the AZ and NZ tissues.

### Changes in Endo-β-mannanase Protein Content during Petiole Abscission in Explants

To examine the expression patterns of endo-β-mannanase at the protein level, *GmMAN1* was heterologously expressed in *E.coli* using the pMAL expression system, and polyclonal antibodies were raised against the fusion protein.

**Figure 5:**
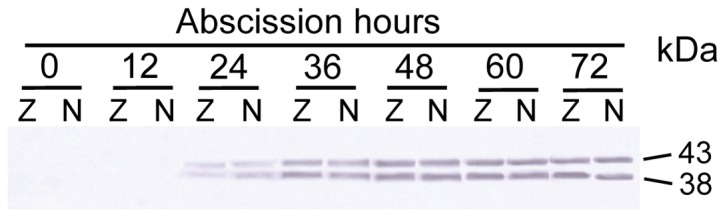
Changes in the abundance of GmMAN1 in abscission zone (Z) and non-abscission zone (N) in soybean petiole explants. Molecular masses (kDa) are indicated on the right. Equal amounts (100 µg) of protein were loaded on each lane.

The presence of endo-β-mannanase protein in the AZ and NZ tissues of soybean petiole explants was measured by protein gel blot analysis with the antibody raised. As shown in Figure 5, up to 12 h, no protein band was detectable in the two tissues. However, 24 h after excising, two protein bands of close molecular mass were detected in both the AZ and NZ tissues. The molecular mass of one band is 43 kDa, identical to that of the deduced mature protein GmMAN1. Another band with a molecular mass of 38 kDa was also detected. This is probably the polyclonal antibodies cross-reacted with another isoform of soybean endo-β-mannanase with high sequence identity to GmMAN1.The intensity of the two bands increased slightly from 24 to 36 h and then remained at that level through 72 h. Again, no difference of band intensity was found between the AZ and NZ tissues at each time point during abscission.

### Changes in Endo-β-mannanase Expression during Leaf Abscission Artificially Induced by Ethephon in Intact Plants

The above results indicate that the increases in endo-β-mannanase activity, isoform numbers, amounts of transcripts and proteins of *GmMAN1* were not restricted to the AZ in soybean petiole explants. Therefore, these changes are not likely to be associated exclusively with abscission. But there remains the question as to why there are these changes. One reason could be that during the preparation of the petiole explants, there is a response to the mechanical wounding imposed by cutting on the stem and petiole (Figure 1).

**Figure 6:**
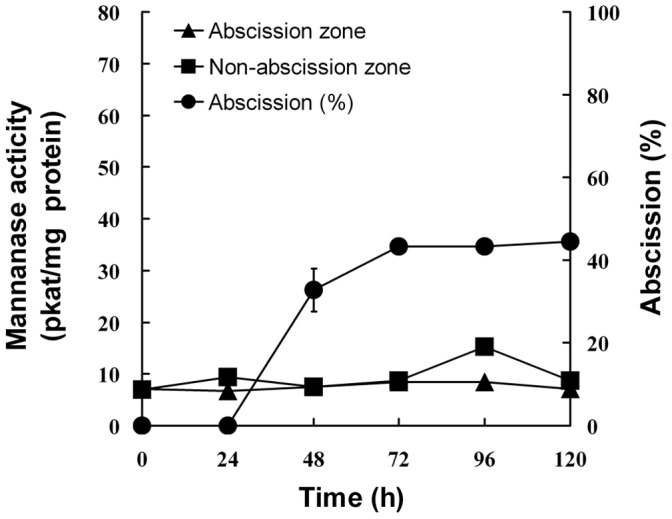
Changes in the cumulative abscission percentage and endo-β-mannanase activity in abscission and non-abscission zones in intact soybean plants treated with 0.1% (v/v) ethephon. Means of three measurements ±SD.

**Figure 7:**
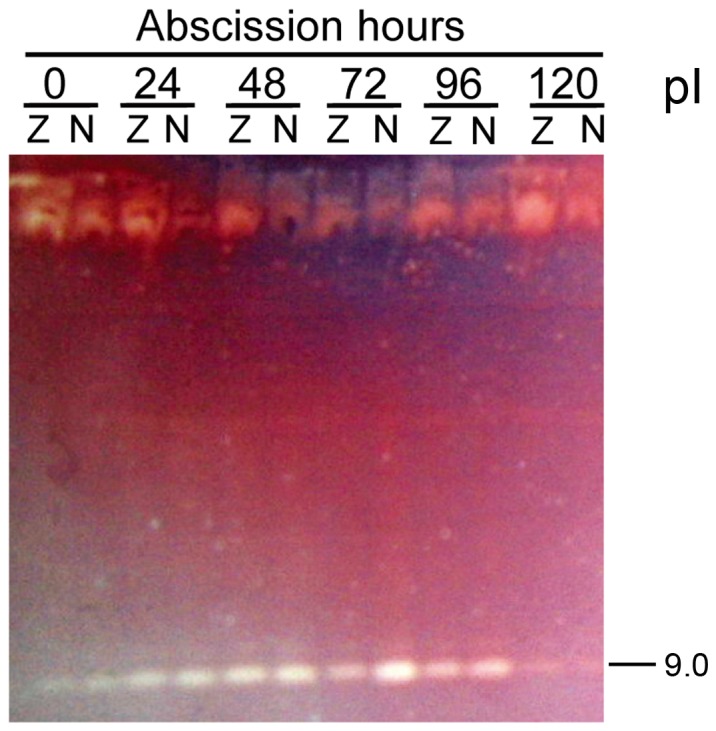
Changes in the ultrathin-layer isoelectric focusing isoform profiles of endo-β-mannanase in abscission zone (Z) and non-abscission zone (N) in intact soybean plants treated with 0.1% (v/v) ethephon. Isoelectric point (pI) is indicated on the right. Equal amounts (30 µg) of protein were loaded on each lane.

In order to eliminate the influence of mechanical wounding (cutting) on the changes in endo-β-mannanase during abscission, intact soybean plants (32-d-old) were artificially induced to abscise leaves by spraying 0.1% (v/v) ethephon. Intact plants sprayed with water were used as a control. It was found that in the control plants, the leaves did not abscise, and a same level of endo-β-mannanase activity (c.a. 9 pkat/mg protein) was detectable in both the AZ and NZ tissues through the experimental period (data not shown). However, in ethephon sprayed intact plants, the leaves began to abscise 24 h after the treatment (Figure 6). The cumulative abscission percentage increased to 44% at 72 h and remained at that level thereafter. Endo-β-mannanase activity in both the AZ and NZ tissues after ethephon treatment was similar with that in control plants, and did not increase during the abscission process. The isoform profiles of endo-β-mannanase during leaf abscission artificially induced by ethephon in intact plants are shown in Figure 7. Only one isoform with a pI of 9.0 was detectable, again with no difference between the AZ and NZ tissues.

Protein and RNA gel blot analysis detected no protein or *GmMAN1* transcripts in either the AZ or NZ tissues during leaf abscission induced by ethephon treatment in intact soybean plants (data not shown).

### Changes in Endo-β-mannanase Expression Patterns in Soybean Plants after Wounding

A wounding experiment was carried out by removing half of the leaf blade of the first pair of true leaves in 17-d-old soybean plants. After wounding, the roots, stem and remaining leaf blades were harvested at 12 h intervals for enzyme and RNA extraction. Endo-β-mannanase activity, isoforms, protein and mRNA contents were analyzed.

**Figure 8:**
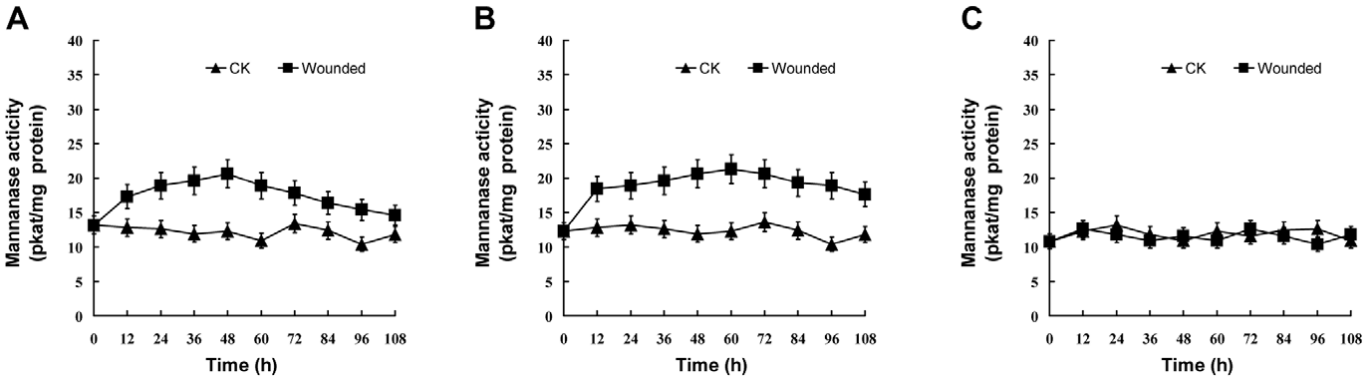
Changes in endo-β-mannanase activity in the leaves (A), stem (B) and roots (C) in soybean plants after wounding. Plants of 17-d-old were wounded by removing half of the leaf blade of the first pair of true leaves. CK: control, unwounded plants. Means of three measurements ±SD.

A similar low endo-β-mannanase activity was present in the roots, stem and remaining leaf blades of unwounded control plants and did not change greatly during the whole experimental period (Figure 8). After the first pair of true leaves were wounded, enzyme activity in the leaves and stem increased, reaching a maximum between 48–60 h after wounding and declining slightly thereafter, but still higher than that of the control plants. However, enzyme activity in the roots did not increase in the wounded plants.

**Figure 9:**
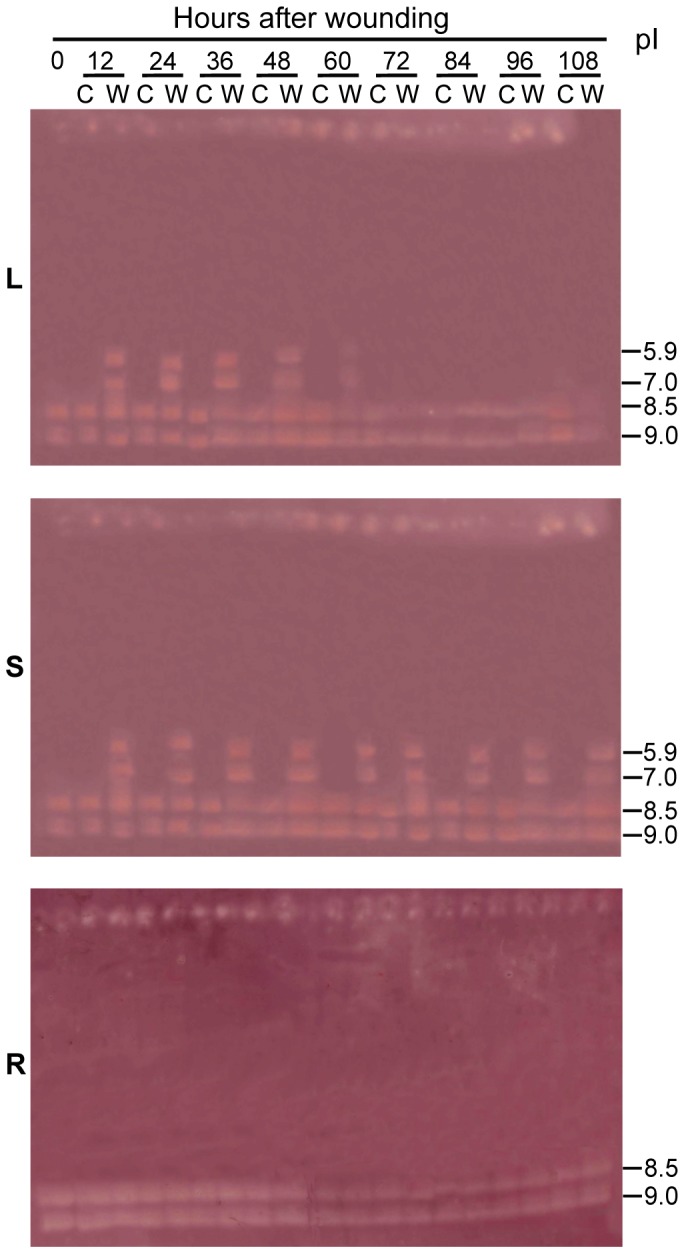
Changes in the ultrathin-layer isoelectric focusing isoform profiles of endo-β-mannanase in the leaves (L), stem (S) and roots (R) in soybean plants after wounding. Plants of 17-d-old were wounded (W) by removing half of the leaf blade of the first pair of true leaves. C: control, unwounded plants. Isoelectric points (pI) are indicated on the right. Equal amounts (30 µg) of protein were loaded on each lane.

Two endo-β-mannanase isoforms with pIs of 9.0 and 8.5 were detectable in the roots, stem and leaves of the unwounded (control) plants (Figure 9) and remained unchanged throughout the experimental period. However, 12 h after wounding, two new endo-β-mannanase isoforms with pIs of 7.0 and 5.9 were detected in the leaves and stem. These were no longer present in the leaves at 72 h after wounding, whereas in the stem they remained at least until 108 h. No new isoforms were detected in the roots of wounded plants compared to the control plants.

**Figure 10:**
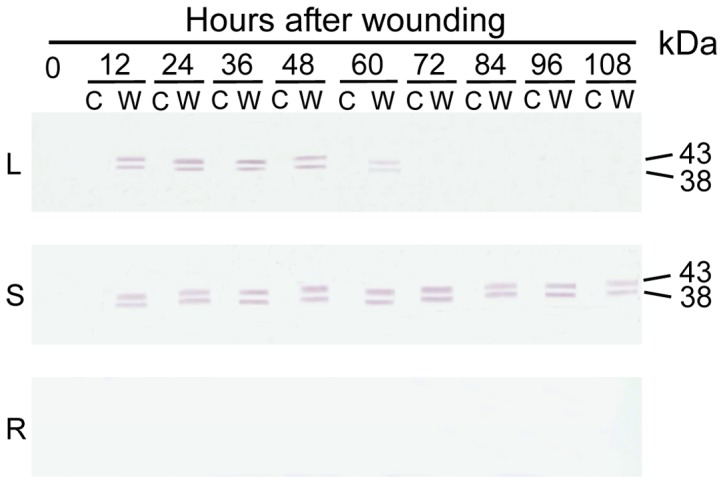
Changes in the abundance of GmMAN1 in the leaves (L), stem (S) and roots (R) in wounded (W) and control (C) soybean plants. Molecular masses (kDa) are indicated on the right. Equal amounts (100 µg) of protein were loaded on each lane.

Western blotting analysis showed that no endo-β-mannanase protein encoded by *GmMAN1* was detectable in the roots, stem or leaves of the control plants (Figure 10). However, 12 h after wounding, two protein bands with molecular masses of 43 and 38 kDa were present in the stem and leaves. These were no longer present in the leaves at 72 h after wounding, but were detectable up to 108 h in the stem. No endo-β-mannanase protein encoded by *GmMAN1* was detected in the roots in wounded plants.

**Figure 11:**
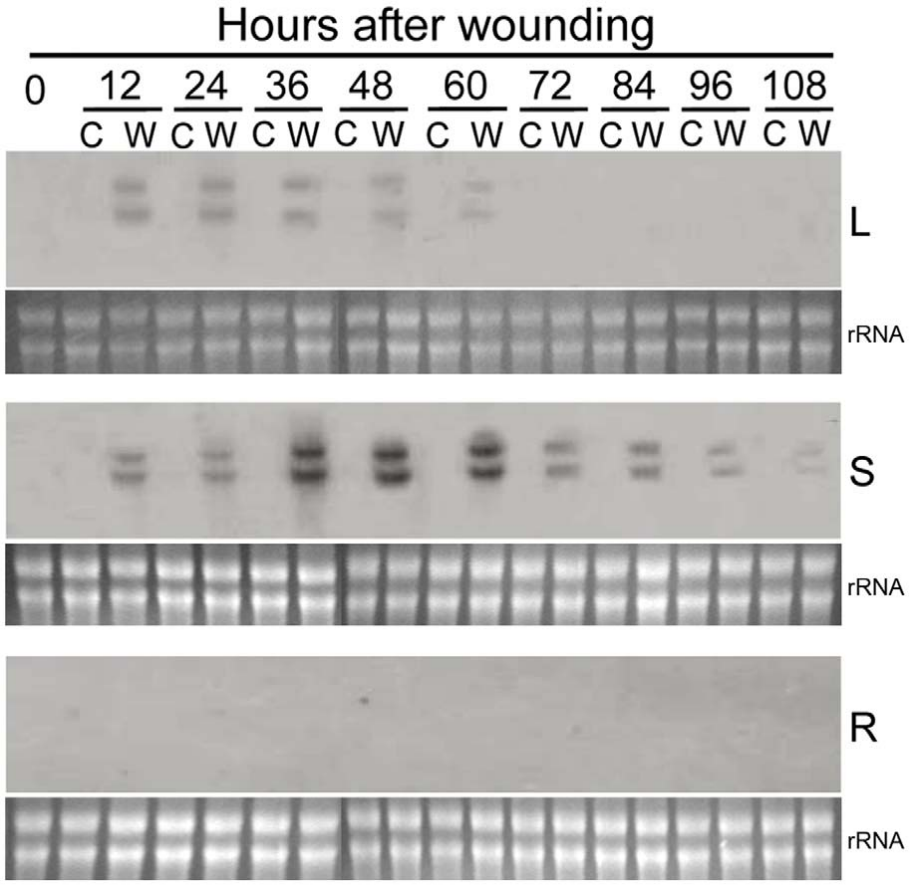
Changes in the abundance of *GmMAN1* transcripts in the leaves (L), stem (S) and roots (R) in wounded (W) and control (C) soybean plants. Equal amounts (20 µg) of RNA were loaded on each lane.

The transcripts of *GmMAN1* were not detectable in the roots, stem and leaves in the unwounded soybean plants (Figure 11). However, two transcripts were detected in the stem and leaves 12 h after the plants were wounded. The intensity of the two bands in the leaves declined gradually until they were no longer present at 60 h after wounding, those in the stem reached a maximum between 36–60 h after wounding and declined gradually thereafter, but still were visible at 108 h. The transcripts of *GmMAN1* could not be detected in the roots in wounded soybean plants.

## Discussion

Abscission is the process by which plant organs are shed, including leaves, flowers and fruits. It includes a complex of biochemical and molecular events, such as de-novo synthesis of cell wall hydrolases leading to the breakdown of cell wall, specifically in only a few layers of cells of the petiole, i.e. the AZ tissues. A number of cell wall hydrolases are involved in abscission and are expressed specifically in the AZ but not in the NZ. For example, the expression of a cellulase mRNA is limited to one or two cell layers on either side of the fracture plane in nonvascular tissues and up to 3 mm distal to the fracture plane in cells within the vascular bundle [40]. The accumulation of an mRNA encoding a cellulase is primarily restricted to the AZ of *Sambucus nigra* [14]. The expression of polygalacturonase activity and genes increases specifically in the AZ during the shedding of leaves and flowers [18], [20]. An increase in expansin activity and accumulation of its transcripts was detected specifically in the AZ of *Sambucus nigra* [24]. Hong *et al.* showed that a GUS-reporter gene driven by the promoters of two tomato abscission polygalacturonase genes is specifically expressed in the AZ [41]. In this study we describe the expression patterns of endo-β-mannanase, another cell wall hydrolase, during soybean leaf abscission. Although the activity and isoform numbers of this enzyme increase, and an endo-β-mannanase gene *GmMAN1* and its encoded proteins are expressed in the AZ tissues in petiole explants during abscission, all these changes also occur in the NZ tissues (Figure 2,3,4,5). Therefore, these changes in endo-β-mannanase are not uniquely associated with abscission.

However, endo-β-mannanase activity and isoform numbers do increase in soybean explants, which may be the result of them being wounded during the preparation of the petiole explants. In support of this: 1) There is no increase in enzyme activity during leaf abscission artificially induced by ethephon treatment in intact plants, where no wounding occurs (Figure 6); 2) When the first pair of true leaves of soybean plants are wounded, there is an increase in enzyme activity, appearance of new isoforms, and expression of *GmMAN1* in the stem as well as in the leaves. In unwounded control plants, only basal enzyme activity and isoforms are detectable, but the transcripts of *GmMAN1* is not detecable by gel blot analysis (Figure 8,9,10,11).

The changes in endo-β-mannanase occurred not only in the local leaves where wounding was applied, but also in the stem which is away from the wounding site (Figure 8,9,10,11). The response of plants to wounding includes local changes involved in wound-healing and also systemic signalling at remote sites [7]. It is suggested that mannooligosaccharides may function as signaling molecules in the non-specific defence response against virus infection in cucumber [31]. Therefore, the changes in endo-β-mannanase in the stem of soybean plants may be induced by mannooligosaccharides produced in the wounded leaves and likely transmitted to the stem via the phloem.

The use of explants was considered as a significant advance in the methodology to study abscission [42]. However, mechanical wounding is inherent in the preparation of explants. Wounding also induces a series of responses, including the synthesis of cell wall hydrolases [7], [27]–[30], although this effect of wounding on explants, or more specifically on the AZ, has sometimes been ignored. Some studies have only investigated the changes in cell wall hydrolases in the AZ, not describing whether these also occurred in the NZ [43]. Others show an increase in activities of several cell wall hydrolases and expression of their genes in the NZ of several plants [15], [44], [45]. Therefore, it is possible that an increase in the activity of some cell wall hydrolases in the AZ described previously is due to wounding.

Multiple isoforms of endo-β-mannanase were detected in soybean plants (Figure 3, 9), implying that this enzyme is encoded by a relative large gene family. The full genome sequence of soybean has recently been released [46], and from this genome sequence Lin *et al.* identified 21 putative endo-β-mannanase genes [37]. In this study, an endo-β-mannanase isoform with a pI of 9.0 was found to occur throughout in the plant, including the AZ and NZ tissues in petiole explants and intact plants, the leaves, stem and roots of wounded and control plants. It may account for the basal enzyme activity in freshly-excised explants and intact plants. Isoforms of pI 7.0 (identical to that of the deduced mature protein GmMAN1) and 5.9 are present only in the AZ and NZ tissues in petiole explants and in the leaves and stem of wounded plants (Figure 3, 9). Therefore, these two isoforms are probably induced by wounding and account for the observed increase in enzyme activity. More isoforms (pIs of 5.5, 5.1, 4.7 and 4.3) are induced in the AZ and NZ in petiole explants than in the leaves and stem of wounded plants. The reason is not clear, but implies that the changes that occur in petiole explants (subjected to both abscission and wounding) are more complex than in the wounded plants.

Two protein bands are detectable in the AZ and NZ tissues in petiole explants and in the leaves and stem of wounded plants using the antibody raised against GmMAN1 (Figure 5, 10). One of the two proteins has a molecular mass of 43 kDa, identical to that of the deduced mature protein GmMAN1; the other has a molecular mass of 38 kDa. Thus the antibody may cross-react with another member of the soybean endo-β-mannanase family, since two mRNAs of close molecular mass are also detectable in the AZ and NZ in petiole explants and in the leaves and stem of wounded plants (Figure 4, 11). Other hydrolases also exhibit similar homologies, e.g. three mRNAs of tomato abscission-related polygalacturonase show similar tissue-specific expression patterns in leaf and flower AZ [45], and three cellulase isoforms occur in tomato flower AZ [44]. The probe used in this study is at 3′-end of the open reading frame of *GmMAN1*, including an 18-bp 3′-UTR, and it could have recognized other members of the soybean endo-β-mannanase family with high sequence identity to this gene. Thus two wounding-inducible endo-β-mannanase genes may exist in soybean plants, with similar expression patterns in the response to wounding.

In conclusion, the expression of *GmMAN1* in the AZ tissues in soybean petiole explants may not be associated with abscission, but is more likely be involved in the response to wounding.

## Supporting Information

Figure S1 The cDNA sequence of soybean endo-β-mannanase *GmMAN1*. The open reading frame is in capital letters. The primers used for the cloning of cDNA are underlined, and the probes used for RNA gel blot analysis are dotted.
(DOC)
