# Supplementary material for: Soybean Endo-β-Mannanase GmMAN1 Is Not Associated with Leaf Abscission, but Might Be Involved in the Response to Wounding
Source: PLoS One. 2012 Nov 16;7(11):e49197. doi: 10.1371/journal.pone.0049197 (PMC3500276; doi:10.1371/journal.pone.0049197)
Supplement: Figure S1 — The cDNA sequence of soybean endo-β-mannanase GmMAN1. The open reading frame is in capital letters. The primers used for the cloning of cDNA are underlined, and the probes used for RNA gel blot analysis are dotted. (DOC) [file pone.0049197.s001.doc]

Supplemental Figure S1

ataaagtatattctcttctctgccgtccaagctagtcgttctctctgtctcgtttatattttttggtttattatattcttctgtttgcacgaagcacactATGGGTTGGAAAGGAGGTCTCAAAAACGTGGTCACCATGTTTACTTTCATGGTGGCCTTGGTTGTTGTTCAACATGTGAAGTGTGGGGACTCAGCTCGCATGTTACTTCAGCACGGTGGCTTCGTTCAACGAAGTGGCACCAACTTCGTTCTGAGTAACAGGCATTTCTACTTCAACGGATTCAACGCGTACTGGTTAATGTACATGGCATCTGACCCAGCCACAAGGCCCAAGGTCACTGCCGTTTTGCAACAAGCTTCTAGCCATGGCTTAACTGTTGCTAGAACTTGGGCTTTCAGTGATGGAGGTTATAGAGCCCTTCAGGTTTCTCCTGGTTCCTACGACGAGAAAGTATTCAGGGGATTGGACTTTGTAGTATCAGAAGCGGGAAAATATGGGGTGCGTTTGATACTGAGCTTGGTGAACAACTGGAAAGATTTTGGTGGCAAAAATCAGTACGTACAGTGGGTGAAGGAACATGGACAGTACGTGAACAGTGAAGATGATTTCTTTTCACATCCTATTGCTAAGCAACATTACAAAAACCATATTAAGGCTGTGTTGACAAGAAAAAACACAATAACTGGGGTGGCATATAAGGACGATCCTGCCATATTTGCGTGGGAACTTATCAATGAACCCCGTTCCCAACACGACAACTCCGGAAAAGTTATTCAGCAATGGGTGATTGAGATGGCTGCCTACGTCAAATCCATCGACAACAATCATTTGTTAGAAATAGGACTCGAAGGGTTCTATGGTGAAACAATGCCAGAGAAAAAACAATTCAATCCTGGGTACCAACTTATTGGCACTGATTTCATTTCTAACAACCTAGTTCACCAAGTTGATTTTGCTACCATGCATCTCTACCCTGAACAATGGTTGCCAGGCTCAAACGAAGCTGCTCAGGTTGCATTTGTTGACAAATGGTTACAAACACACATTCAAGATGCCAAAAATGTTCTGGGGAAGCCTATTGTTGTTGGTGAGTTTGGCAAGTCTTCGAAGTCATATAGTGTGGTTGAAAGGGACAATTACCTGAGCAAAATGTATAATGCCATATACAGTAGTGCTAGTAGTGGGGGACCCTGTGCTGGTGGGCTTTTTTGGCAGCTCATGGCTAAAGGAATGGATGGTTTACGTGATGGTTATGAAGTCATCTTTGAGGAGAGTCCTTCAACTACCAGAATTATAGATCAACAATCCCACAAAATGTCAAGTATTGCTTAGtgaacaagcattaatgtcgcctacctagctcatgtaaatatatatgtaacaaacttatagtttgtttggttacttatatattcctcccattacaagcactattcatag
